# Global Prevalence of Deep Vein Thrombosis in Patients with Spinal Cord Injury: A Systematic Review and Meta-Analysis

**DOI:** 10.1089/neur.2024.0144

**Published:** 2025-06-12

**Authors:** Kai Wang, Kunbin Li, Baochao Fan, Yingchun Gu, Xiaopeng Wen, Zhiyuan Wu, Xianli Yao, Pingge Sun, Bing Jiao, Xiaoxing Li, Yage Liu, Liming Lu

**Affiliations:** ^1^ Department of Neurological Rehabilitation, Zhengzhou Central Hospital Affiliated to Zhengzhou University, Zhengzhou, China.; ^2^ Clinical Research and Big Data Laboratory, South China Research Center for Acupuncture and Moxibustion, Medical College of Acu-Moxi and Rehabilitation, Guangzhou University of Chinese Medicine, Guangzhou, China.; ^3^ Department of Cardiac Rehabilitation, Zhengzhou Central Hospital Affiliated to Zhengzhou University, Zhengzhou, China.

**Keywords:** analysis, deep vein thrombosis, global prevalence, spinal cord injury

## Abstract

A meta-analysis of deep vein thrombosis (DVT) in patients with spinal cord injury (SCI) was performed using five databases (PubMed, Embase, the Cochrane Library, Web of Science, and Scopus) from 2000 to March 2023. Observational descriptive studies investigating the prevalence of DVT among patients with SCI were included. Data were retrieved by author, country, continent, gender, age, sample source, and additional variables. Risk of bias was evaluated using the Joanna Briggs Institute Critical Appraisal Instrument for Studies Reporting Prevalence. Data and random-effects models were used to synthesize existing findings. Among 45 studies, the overall pooled estimated prevalence of DVT was 14.53% (95% confidence interval [CI], 11.22 − 17.84%) in patients with SCI (*n* = 87,294), including 14.77% (95% CI, 11.19 − 18.35%) in patients with acute SCI and 19.02% (95% CI, 11.51 − 26.53%) in patients with SCI older than 18 years. A total of 26 studies from hospitals showed that the combined prevalence estimate of DVT in patients with SCI was 16.41% (95% CI, 11.36 − 21.45%), and in 19 studies from rehabilitation institutions was 12.33% (95% CI, 8.25 − 16.42%). Moreover, the prevalence of DVT in patients with SCI is influenced by factors such as regional distribution, demographic characteristics, the extent of nerve damage, the level of the lesion, and the implementation of thromboprophylaxis. We estimated the overall pooled prevalence of DVT after SCI in distinctive characteristics. These findings can provide a reference for future epidemiological studies of DVT in patients with SCI. Given the substantial variety of the included studies (e.g., diagnostic methodologies, demographic characteristics), our results should be interpreted with caution.

## Introduction

Spinal cord injury (SCI) causes a temporary or permanent loss of motor, sensory, and/or autonomic nerve function, depriving patients of some or all of their ability to work, move around, and care for themselves. The average lifetime cost of treating an individual with traumatic SCI is between US$500,000 and $2 million, depending on factors such as extent and location of injury (serious injuries correlate with increased disability and costs). Total direct costs of caring for individuals with SCI exceed $7 billion per year in the United States. The cause of SCI could be traumatic or nontraumatic, which includes infection, toxins, tumor, degenerative disease, inflammation, and vascular and congenital causes.^1^ Deep vein thrombosis (DVT) occurs when abnormal clotting develops in the deep veins. Thrombus shedding can cause pulmonary embolism (PE). DVT and PE are collectively referred to as venous thromboembolism (VTE), which are manifestations of the same disease at various stages. Patients with SCI who have experienced trauma, inactivity, paralysis, or surgery are at high risk for DVT and PE due to the simultaneous presence of Virchow’s risk: stasis, vessel wall damage, and hypercoagulability.^2^ DVT is one of the most common and severe complications during the acute stage of SCI; it may be asymptomatic, but its eventual PE is the third-most prevalent cause of mortality in patients with SCI.^3^

Several previous studies reported that the occurrence rate of DVT in SCI ranges from 40% to 100%, considering that population demographics and study design are different in each study.^4–10^ Other studies suggested that the estimated incidence of DVT in patients with SCI ranged from 5% to 26%.^11–13^ A number of studies have consistently reported the predictors that may increase the risk of DVT in patients with SCI, including age, gender, thromboprophylaxis, severity and location of the injuries, and comorbidity.^14–19^ It is apparent that a meaningful dialogue on the necessity of DVT testing in patients with SCI depends on understanding the expected rate of DVT occurrence in these individuals. However, there is a shortage of reliable data on this issue, and the reported prevalence rates of DVT vary greatly. Therefore, we selected articles published between the year 2000 and March 2023, and conducted a meta-analysis to analyze the global prevalence of DVT in SCI and its association with populations from different regions, gender, nerve defect degree, sample source, gender, and thromboprophylaxis.

## Methods

### Search strategy

The literature search was performed across five databases (PubMed, Embase, the Cochrane Library, Web of Science, and Scopus) from 2000 to March 2023, encompassing global studies. This meta-analysis was performed according to the Preferred Reporting Items for Systematic Reviews and Meta-Analyses (PRISMA) guidelines. The search terms included “(spinal cord injury or spinal cord trauma) and (venous thrombosis or deep vein thrombosis) and (epidemiology or prevalence or incidence).” A list of references for all pertinent reviews and acceptable publications was also meticulously examined. The search strategy is provided in the appendix in Supplementary Data.

### Study selection

#### Types of studies

Studies that qualify for inclusion in our analysis adhered to the following criteria: (1) studies published after the year 2000; (2) observational studies with a cross-sectional, case–control, or cohort design; (3) studies reporting the prevalence of DVT in patients with SCI; (4) target population was hospitalized patients or from the rehabilitation facilities; and (5) the publication language is English, and articles have been published. The exclusion criteria for studies were as follows: (1) studies in the form of reviews, case reports, letters, conference abstracts, protocols, or animal experiments; (2) studies that did not report sufficient data about the incidence or prevalence of DVT among patients with SCI and failed to contact the authors via email; (3) full text not available; (4) when multiple articles evaluated the same study cohort, the larger study was included; when the same author published with the same cohort, we selected the most recent publication year; (5) nonobservational studies; and (6) studies with low quality as measured by a quality assessment. To mitigate the influence of selection bias on the findings, all observational studies that satisfied the inclusion criteria were incorporated into the analyses. Therefore, there is no requirement for sample size thresholds. However, validated diagnostic methods are necessary.

#### Types of participants

All patients included in our study were diagnosed with SCI (including those with traumatic and nontraumatic, acute and nonacute, at any age, with or without anticoagulant therapy). The diagnosis of SCI is based on clinical presentation and is confirmed by radiological evaluation, including computed tomography and magnetic resonance imaging. The target population comprised hospitalized patients or those from rehabilitation facilities, with no classification limits for hospitals and rehabilitation institutes.

#### Types of exposure

We sought to identify all studies that screened for DVT in asymptomatic or symptomatic patients with SCI who were assessed by the color Doppler ultrasound or venography.

### Data extraction and quality assessment

The articles were screened by reading the title and abstract to find the relevant article and then the eligible studies were selected by full-text review. Two independent investigators (K.W. and X.W.) performed data extraction from the included studies. The extracted data were then standardized using Excel software, and any unstandardized data were processed accordingly before mutual verification. Any discrepancies were resolved by consensus. The data collection encompassed various parameters such as the author, country, continent, study period, publication year, gender, age, sample source, type of sample, thromboprophylaxis methods, the number of participants, and the incidence of DVT, along with a detailed account of gender distribution among the participants. We inserted the above data into one table (Table 1).

**Table 1.** Characteristics of the Included Studies

Source and study period	Continent	No.		Thrombosis rate	Study age inclusion, y	Sample source	Type of sample	VTE prophylaxis measures
		DVT	Total					
Aito et al., 2002^12^ (Italy) January 1, 1996 to May 15, 2000	Europe	48 Male: 45 Female: 3	275 Male: 221 Female: 54	17.5%	All	Regional Spinal Unit	Acute SCI	Mechanical and pharmacological prophylaxis
Maxwell et al., 2002^20^ (USA) January 1995 to December 1999	North America	10	111 Male: 90 Female: 21	9.0%	15–91	Level I trauma center	Acute TSCI	Mechanical and pharmacological prophylaxis
McKinley et al., 2002^21^ (USA)	North America	21	117 Male: 89 Female: 28	17.9%	≧18	A Level I trauma center	NTSCI and TSCI	NA
Green et al., 2003^22^ (USA) September 1992 to December 1995	North America	51 Male: 35 Female: 16	243 Male: 193 Female: 50	21.0%	All	SCI Unit at the Rehabilitation Institute	Acute SCI	NA
Kadyan et al., 2003^23^ (USA) January 1, 1996 to December 31, 1998	North America	12	92 Male: 68 Female: 24	13.0%	15–86	Acute rehabilitation center	TSCI	NA
Riklin et al., 2003^24^ (Switzerland) January 1, 1998 to December 31, 2000	Europe	27 Male: 18 Female: 9	1209 Male: 887 Female: 322	2.2%	5–90	Swiss Paraplegic Centre	TSCI and NTSCI	Mechanical and pharmacological prophylaxis
Hebbeler et al., 2004^25^ (USA) June 2000 to June 2002	North America	2	129	6.9%	All	Rehabilitation hospital	Acute SCI	Mechanical and pharmacological prophylaxis
Furlan et al., 2005^26^ (Canada) January 1998 to December 2000	North America	2 Male: 0 Female: 2	55 Male: 38 Female: 17	3.6%	17–90	Neuroscience Centre	Acute cervical TSCI	NA
Saraf et al., 2007^27^ (India) July 2002 to December 2005	Asia	7	70 Male: 59 Female: 11	10.0%	16–59	Hospital	Acute SCI	No prophylaxis
Tauqir et al., 2007^28^ (Pakistan) October 10, 2005 to December 10, 2005	Asia	3	194 Male: 50 Female: 144	1.5%	16–39	Hospital and Rehabilitation Center	TSCI	NA
Rathore et al., 2008^13^ (Pakistan) October 9, 2005 to November 9, 2005	Asia	9 Male: 5 Female: 4	187 Male: 80 Female: 107	4.8%	3–75	Hospital and rehabilitation center	Acute TSCI	NA
Worley et al., 2008^29^ (Canada)1994–2004	North America	4	90 Male: 79 Female: 11	4.4%	All	Rehabilitation center	Acute TSCI	Mechanical and pharmacological prophylaxis
Gorman et al., 2009^30^ (USA) January 2002 to December 2003	North America	16	114	14.0%	All	Rehabilitation center	Acute SCI	Mechanical and pharmacological prophylaxis
Sugimoto et al., 2009^31^ (Japan)2005–2008	Asia	11 Male: 10 Female: 1	52 Male: 41 Female: 11	21.2%	19–94	Hospital	Acute cervical SCI	Mechanical prophylaxis only
Arnold et al., 2010^32^ (USA) January 1, 2006 to December 31, 2006	North America	6	24	25.0%	All	Level I regional trauma center	Acute TSCI	Mechanical and pharmacological prophylaxis
Germing et al., 2010^33^ (German) January 2007 to September 2008	Europe	63	139 Male: 88 Female: 51	45.3%	19–90	Hospital	SCI	Mechanical and pharmacological prophylaxis
Chung et al., 2011^34^ (Korea) November 2009 to October 2010	Asia	16 Male: 12 Female: 4	37 Male: 26 Female: 11	43.2%	20–80	Medical center	Acute TSCI and NTSCI	Mechanical prophylaxis only
Maung et al., 2011^35^ (USA) 2007–2008	North America	618	18,302	3.4%	All	the National Trauma Data Bank	TSCI	NA
Wu et al., 2012^36^ (China) December 2018 to November 2011	Asia	7	143 Male: 119 Female: 24	4.9%	18–87	Hospital	Traumatic cervical SCI	NA
Do et al., 2013^9^ (Korea) January 2002 to July 2011	Asia	51 Male: 38 Female: 13	185 Male: 133 Female: 52	27.6%	17–83	Rehabilitation Unit	TSCI and NTSCI	Mechanical prophylaxis only
Pierfranceschi et al., 2013^37^ (Italy) January 2003 to December 2007	Asia	19	94 Male: 80 Female: 14	20.2%	All	Rehabilitation Unit	Acute TSCI	Mechanical and pharmacological prophylaxis
Chung et al., 2014^38^ (China) 1998–2008	Asia	277 Male: 177 Female: 100	47,916 Male: 30,036 Female:17,880	0.6%	All	Taiwan National Health Insurance Research Database	SCI	NA
Guerra et al., 2014 (Brazil)^39^ January 2011 to April 2012	South America	17 Male: 11 Female: 6	100 Male: 72 Female: 28	17.0%	>18	The Physical Medicine and Rehabilitation Institute	TSCI and NTSCI	NA
Masuda et al., 2015^8^ (Japan) April 2007 to December 2012	Asia	22	211	10.4%	All	Spinal Injuries Center	Acute traumatic cervical SCI	Mechanical prophylaxis only
Matsumoto et al., 2015^40^ (Japan) November 2012 to June 2013	Asia	12 Male: 11 Female: 1	29 Male: 25 Female: 4	41.4%	25–78	Hospital	Acute TSCI	NA
Zhu et al., 2015^41^ (China) January 2013 to December 2013	Asia	46	143 Male: 95 Female: 48	32.2%	All	Hospital	Acute TSCI	NA
Hoh et al., 2016^42^ (USA) 2002–2010	North America	407	10,669 Male: 7761 Female: 2900	3.8%	All	Nationwide In-Patient Sample database	Traumatic cervical SCI	NA
Joseph et al., 2016^43^ (South Africa) September 15, 2013 to September 14, 2014	Africa	8	141 Male: 124 Female: 21	5.7%	>18	Hospital	Acute TSCI	NA
Mackiewicz et al., 2016^44^ (Poland) 2007–2009 and 2011–2013	Europe	5 Male: 4 Female: 1	63 Male: 48 Female: 15	7.9%	13–65	Rehabilitation Department	Chronic SCI	NA
Piran et al., 2016^45^ (Canada) 2009–2015	North America	7	151 Male: 106 Female: 45	4.6%	17–91	Hospital	Acute SCI	NA
Wang et al., 2016^46^ (China) 2013–2014	Asia	55 Male: 22 Female: 23	279 Male: 164 Female: 115	19.7%	43–62	Hospital	Acute SCI	Mechanical prophylaxis only
Clements et al., 2016 (Australia) 2010–2013	Oceania	30 Male: 27 Female: 3	222 Male: 174 Female: 48	13.5%	All	Victorian Spinal Cord Service	Acute TSCI	Mechanical and pharmacological prophylaxis
Groves et al., 2017 (Nepal)^47^ April 25, 2015 to June 16, 2016	Asia	7	117 Male: 65 Female: 52	6.0%	2–86	Spinal Injury Rehabilitation Centre	TSCI	NA
Maharaj et al., 2017^48^ (Australia) January 1, 2001 to December 31, 2012	Oceania	86	384 Male: 328 Female: 56	22.4%	18–91	Hospital	Acute TSCI	NA
Ohbe et al., 2019^49^ (Japan) January 2008 to November 2016	Asia	13	213 Male: 167 Female: 46	6.1%	36–72	Hospital	Acute TSCI	NA
Hon et al., 2020^50^ (USA) January 1, 2011 to December 12, 2016	North America	31 Male: 25 Female: 6	189 Male: 140 Female: 49	16.4%	16–85	Acute rehabilitation facility	Acute TSCI	NA
Kumagai et al., 2020 (Japan)^51^ January 2011 to April 2017	Asia	20	114 Male: 84 Female: 30	17.5%	17–89	Hospital	Acute SCI	Mechanical prophylaxis only
Wang et al., 2020^52^ (China)2014–2018	Asia	157	3487 Male: 2509 Female: 978	4.5%	18–87	Tertiary trauma center	TSCI	NA
Yu et al., 2020^53^ (China) January 2016 to December 2017	Asia	35 Male: 14 Female: 21	80 Male: 36 Female: 44	43.8%	All	Hospital	TSCI	Partial patients received mechanical prophylaxis only
UII et al., 2021^54^ (German) June 2001 to June 2019	Europe	8	60 Male: 59 Female: 1	13.3%	18.3–48.7	Hospital	Acute TSCI	NA
Zhang et al., 2021 (China) August 1, 2018 to December 12, 2020	Asia	86	250 Male: 186 Female: 6	34.4%	All	Rehabilitation Institute	SCI	NA
Ichikawa et al., 2022^55^ (Japan) January 2011 to April 2017	Asia	9	57 Male: 47 Female: 10	15.8%	4–93	Hospital	Traumatic cervical SCI	Mechanical prophylaxis only
Afsar et al., 2022^56^ (Turkey)2005–2019	Asia	17	252 Male: 175 Female: 77	6.7%	All	In-patient rehabilitation unit	NTSCI and TSCI	NA
Jiang et al., 2022^57^ (China) January 2018 to January 2020	Asia	23 Male: 12 Female: 11	130 Male: 72 Female: 58	17.7%	All	Hospital	TSCI	NA
Lv et al., 2022^58^ (China) January 2018 to December 2021	Asia	38 Male: 27 Female: 11	175 Male: 129 Female: 46	21.7%	All	Regional Spinal Unit	Acute SCI complicated with cervical fractures	NA

DVT, deep vein thrombosis; NTSCI, nontraumatic spinal cord injury; SCI, spinal cord injury; TSCI, traumatic SCI; VTE, venous thromboembolism.

Two researchers (K.W. and Y.G.) independently assessed the risk of bias in the included studies using the Joanna Briggs Institute Critical Appraisal Instrument for Studies Reporting Prevalence Data,^59^ and the assessment was checked by a third reviewer (B.F.) (Supplementary Table S1 in Supplementary Data). There were nine items in total (eMethods in the Supplementary Data). Studies were categorized based on the percentage of “yes” answers as high risk (≤49%), moderate risk (50 − 69%), and low risk (≥70%). Thus, higher total scores indicated better quality and lower risk.

### Statistical analysis

Our data analysis was performed using “Metafor”^60^ and “Meta”^61^ packages implemented in R Statistical Software^62^ with a random-effects model. Forest plots were used to show the results graphically. *I^2^* statistic and the *p* value were used to assess the heterogeneity (Cochrane Q statistic) and to choose the effect model. If *I^2^* ≤ 50% and *p* ≥ 0.1, the pooled studies were considered to be homogeneous, and a fixed-effect model was selected. Otherwise, if *I^2^* > 50% and *p* < 0.1, it indicated that a statistical heterogeneity existed among studies, and a random-effects model was selected. In this meta-analysis, two-sided *p* < 0.05 was considered statistically. For the overall prevalence of DVT in patients with SCI, we used Egger’s test to evaluate the publication bias of the included studies, shown using funnel plots. The sensitivity analysis was used to examine the robustness of the pooled effects of the studies included, taking into account both the quality of the studies and their sample sizes. The inter-rater reliability was assessed between the two independent reviewers, with values greater than 0.75 classified as excellent.

## Results

### Study selection and characteristics

**FIG. 1.**
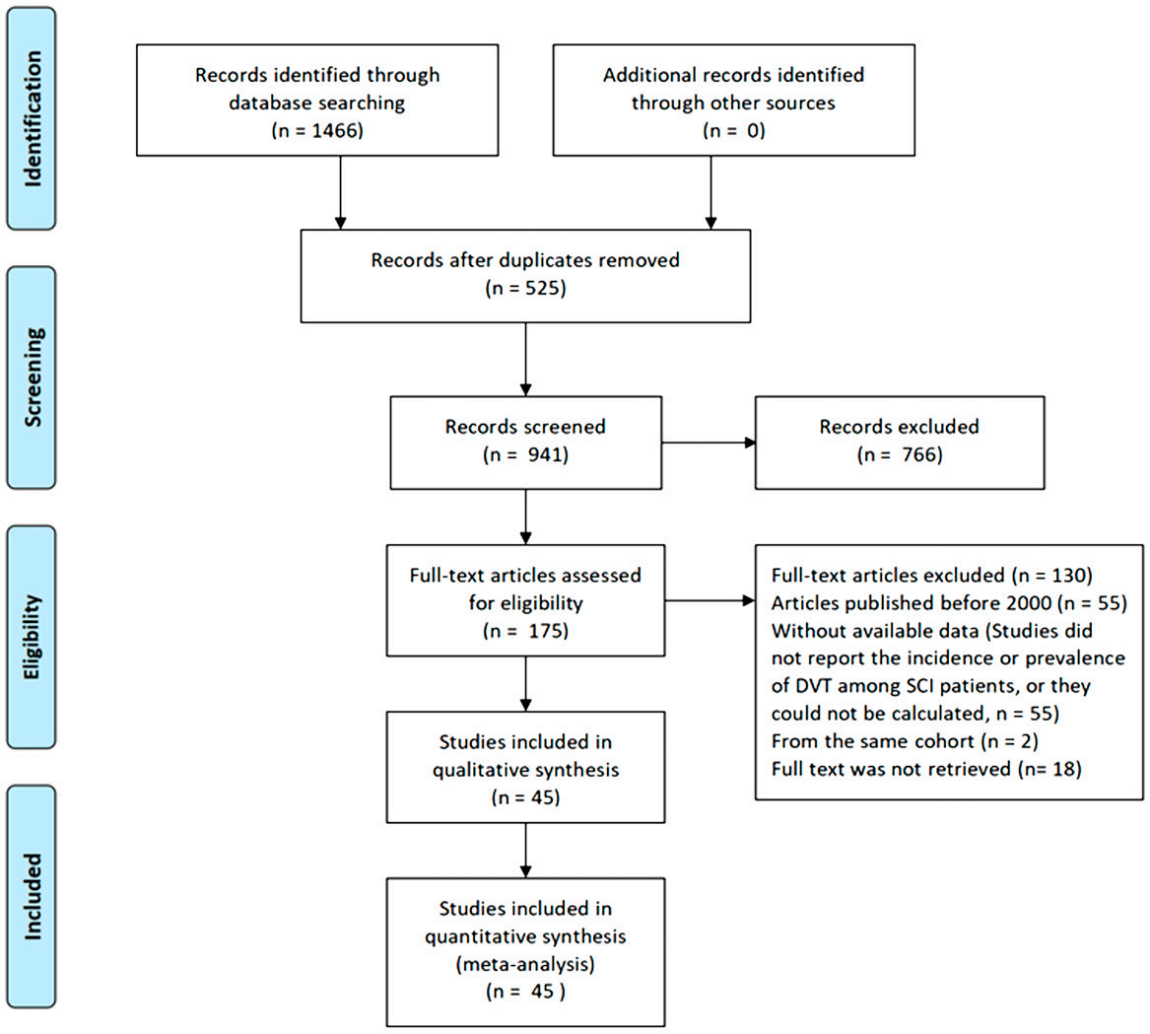
Diagram of literature search and study selection.

A total of 1466 relevant records were initially identified from the five databases. After removing duplicate entries, 525 records were excluded. After screening the titles and abstracts of the studies, 766 of them were excluded. After full-text screening, 45 studies^8,9,12,13,20–22,24,26–31,63^ with 87,294 patients were finally included in the qualitative and quantitative synthesis. The kappa statistic value for the assessment of inter-rater reliability is 0.8. The PRISMA flow diagram is shown in Figure 1.

A total of 45 studies were included, spanning across 16 distinct countries (Table 1). The publication years ranged from 2000 to 2022, with sample sizes varying from 29 to 47,916 participants. Five (11.11%) studies^12,24,33,44,54^ were conducted in Europe, 23 (51.11%) studies^8,9,13,27,28,31,34,36,41,49,51–53,56,63^ in Asia, 13 (28.89%) studies^20–23,25,26,29,30,32,35,42,45,50^ in North America, 2 (4.44%) studies^48,64^ in Oceania, 1 (2.22%) study^39^ in South America, and 1 (4.44%) study^43^ in Africa.

### Pooled and stratified prevalence of DVT in patients with SCI

#### Overall prevalence of DVT in patients with SCI

**FIG. 2.**
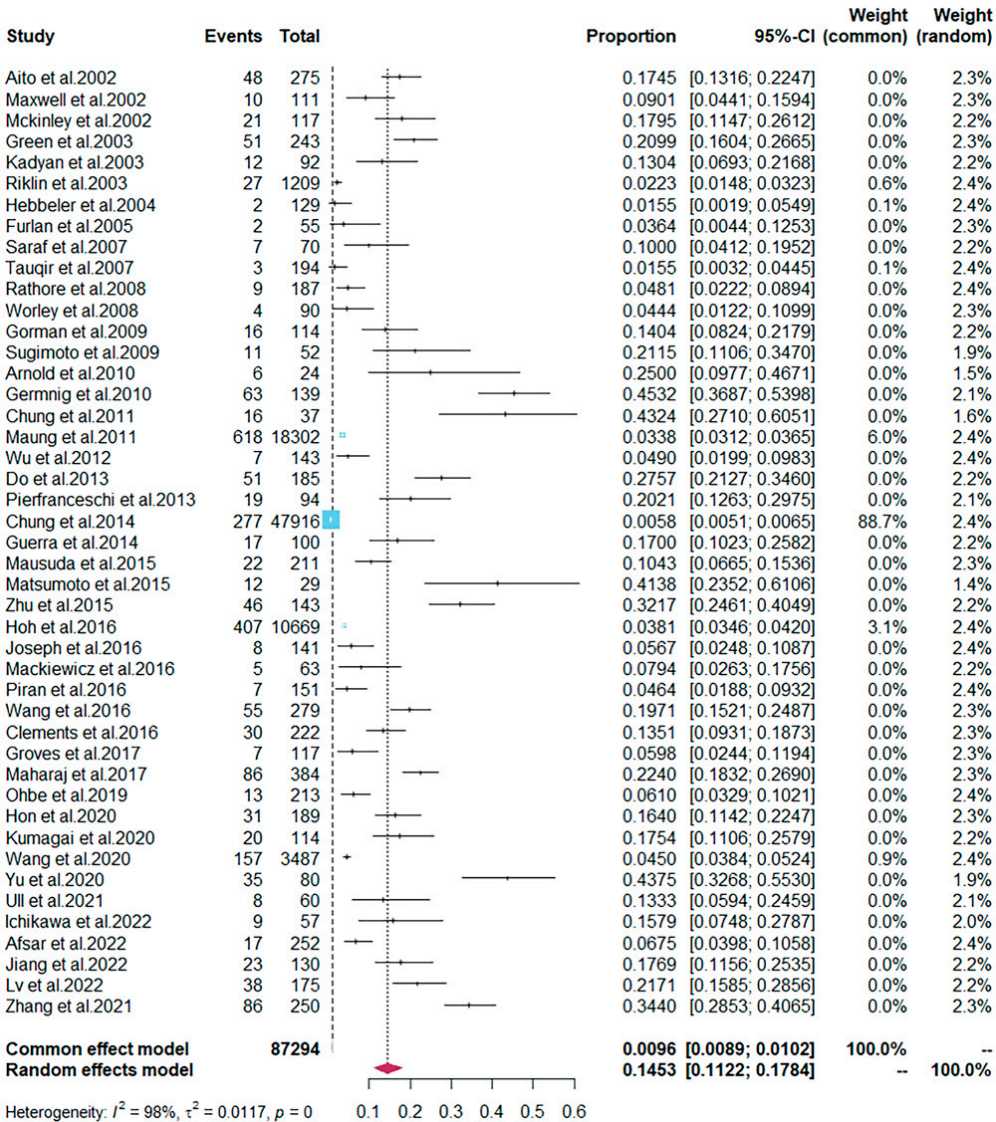
Overall prevalence of deep vein thrombosis (DVT) in patients with spinal cord injury (SCI).

Considering regional variations, gender, age, acute versus chronic SCI, and study quality, the prevalence of DVT in patients with SCI estimated by meta-analysis ranged from 1.55% to 45.32%. The random-effects pooled overall estimated prevalence of DVT in patients with SCI was 14.53% (95% confidence interval [CI], 11.22 − 17.84%) (*I^2^* = 98%, *p =* 0), as shown in Figure 2.

#### Prevalence of DVT after SCI by sample source

We conducted a subgroup analysis based on sample sources to assess the prevalence of DVT after SCI. Twenty-six (57.78%) studies^12,20,26,27,31,32,34–36,41,42,45,48,49,51,63^ from the hospital showed that the combined prevalence estimate of DVT in patients with SCI was 16.41% (95% CI, 11.36 − 21.45%). Nineteen (42.22%) studies^8,9,13,21–25, 28–30,37,39,44,47,50,56,64,65^ from rehabilitation institutions showed that the result was 12.33% (95% CI, 8.25 − 16.42%) (Supplementary Fig. S1 in Supplementary Data).

#### Prevalence of DVT after SCI by continent

Forty-five eligible studies were from six continents, with 5 from Europe (*n* = 1746), 23 from Asia (*n* = 54,415), 13 from North America (*n* = 30,286), 2 from Oceania (*n* = 606), 1 from South America (*n* = 100), and 1 from Africa (*n* = 141). The subgroup analyses demonstrated statistically significant differences by continent (*p* < 0.01). Among the studies, the prevalence of DVT in patients with SCI was 17.02% (95% CI, 2.54 − 31.51%) in Europe, 16.86% (95% CI, 11.63 − 22.10%) in Asia, 9.49% (95% CI, 5.65 − 13.33%) in North America, 18.00% (95% CI, 9.29 − 26.70%) in Oceania, 17.00% (95% CI, 10.23 − 25.82%) in South America, and 5.67% (95% CI, 2.48 − 10.87%) in Africa (Supplementary Fig. S2 in Supplementary Data).

#### Prevalence of DVT after SCI by gender

There were 17 (37.78%) studies^9,12,13,22,24,31,34,38–40,42,44,46,53,57,58,64^ reporting the prevalence rates of DVT in patients with SCI in male and female, and 1 study only observed the prevalence in females. These 18 (40.00%) studies^9,12,13,22,24,26,31,34,38–40,42,44,46,53,57,58,64^ included a total number of 32,515 male (63.2%) and 18,911 female (36.8%). The combined prevalence of DVT among patients with SCI was 16.84% in male (95% CI, 11.34 − 22.34%) (*I^2^* = 95%; *p* < 0.01) and 15.20% (95% CI, 9.33 − 21.07%) in female (*I^2^* = 90%; *p* < 0.01) (Supplementary Fig. S3 in Supplementary Data).

#### Prevalence of DVT after SCI by neurological status

We noted variations in the prevalence of DVT among patients with SCI, categorized by their neurological status. The American Spinal Injury Association (ASIA) impairment scale^66^ was used to evaluate the degree of nerve defect in patients with SCI. A total of 10 (22.22%) studies^9,12,36,39,40,42,44,46,53,57^ (*n* = 1467) included the number of patients in ASIA grade A (*n* = 398), ASIA grade B (*n* = 306), ASIA grade C (*n* = 387), and ASIA grade D (*n* = 376). The pooled prevalence of DVT in patients with SCI with ASIA grade A was 29.50% (95% CI, 15.02 − 43.98%), ASIA grade B 16.83% (95% CI, 10.22 − 23.45%), ASIA grade C 15.06% (95% CI, 9.43 − 20.69%), and ASIA grade D 13.43% (95% CI, 6.68 − 20.18%) (Supplementary Fig. S4 in Supplementary Data).

#### Prevalence of DVT after SCI by lesion level

The prevalence of DVT in patients with SCI varied in different lesion levels. There were 12 (26.67%) studies^8,9,26,31,35,36,39,42–44,55,58^ (*n* = 30,153) that reported the prevalence of DVT in different lesion levels, all of which reported the patients with cervical SCI (*n* = 22,934), only 5 (11.11%)^9,35,39,43,44^ of which reported both thoracic SCI (*n* = 4844) and lumbar SCI (*n* = 2375). The pooled prevalence of DVT in patients with cervical SCI was 9.87% (95% CI, 5.44 − 14.30%), thoracic SCI 13.99% (95% CI, 4.07 − 23.91%), and lumbar SCI 12.51% (95% CI, 0.03 − 24.99%) (Supplementary Fig. S5 in Supplementary Data).

#### Prevalence of DVT after SCI by thromboprophylaxis

A total of 19 (42.22%) studies^9,12,20,24,27,29–32,34,40,51,53,63,64^ provided definitively available data on DVT prophylactic methods. There were 10 (22.22%) studies,^12,20,24,25,29,30,32,33,37,64^ including 2361 patients with SCI undergoing both mechanical and pharmacological prophylaxis, whose pooled prevalence was 14.66% (95% CI, 6.63–22.70%). Nine (20%) studies^9,20,31,34,40,46,51,53,55^ included 1010 patients with SCI undergoing only mechanical prophylaxis, with the pooled prevalence being 19.67% (95% CI, 13.63–25.71%). Only one (2.22%) study^27^ was with no prophylactic methods, and its prevalence of DVT was 10% (95% CI, 4.12 − 19.52%) (Supplementary Fig. S6 in Supplementary Data).

#### Prevalence of DVT in patients with acute SCI

There were 26 (57.78%) studies^8,12,13,20,22,26,27,29–32,34,41,42,45,63^ showing the prevalence of DVT in patients with acute SCI (*n* = 3792). Also, the combined proportion of DVT in patients with acute SCI was 14.77% (95% CI, 11.19 − 18.35%) (Supplementary Fig. S7 in the Supplementary Data).

#### Prevalence of DVT in patients with SCI older than 18 years

Our objective was to investigate the correlation between age and prevalence; however, we encountered challenges in effectively categorizing meaningful age stages. Finally, we got 13 (28.89%) studies^21,31,33^,^34^,^36^,^39^,^40^,^43^,^46^,^48^,^49^,^52^,^54^ on the prevalence of DVT among the patients with SCI older than 18 years (*n* = 5182), and the pooled prevalence rate was 19.02% (95% CI, 11.51 − 26.53%) (Supplementary Fig. S8 in Supplementary Data).

### Publication bias

**FIG. 3.**
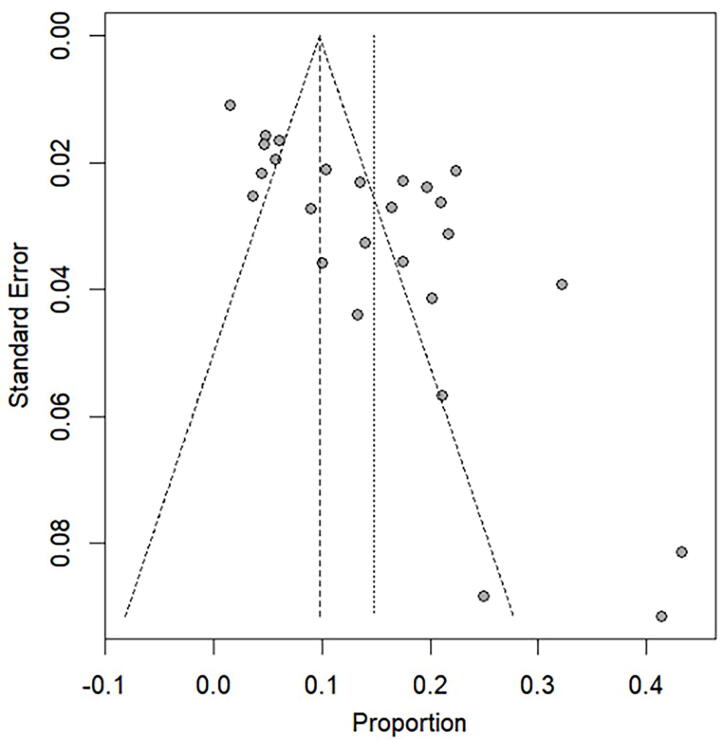
Funnel plot of publication bias.

Among the 45 included studies, the Egger test showed statistically significant publication bias (*p* < 0.05). Consequently, the bias in publication may serve as the origin of heterogeneity (Supplementary Fig. S9). In addition, the funnel plot shows a similar result, as shown in Figure 3.

### Sensitivity analysis

**FIG. 4.**
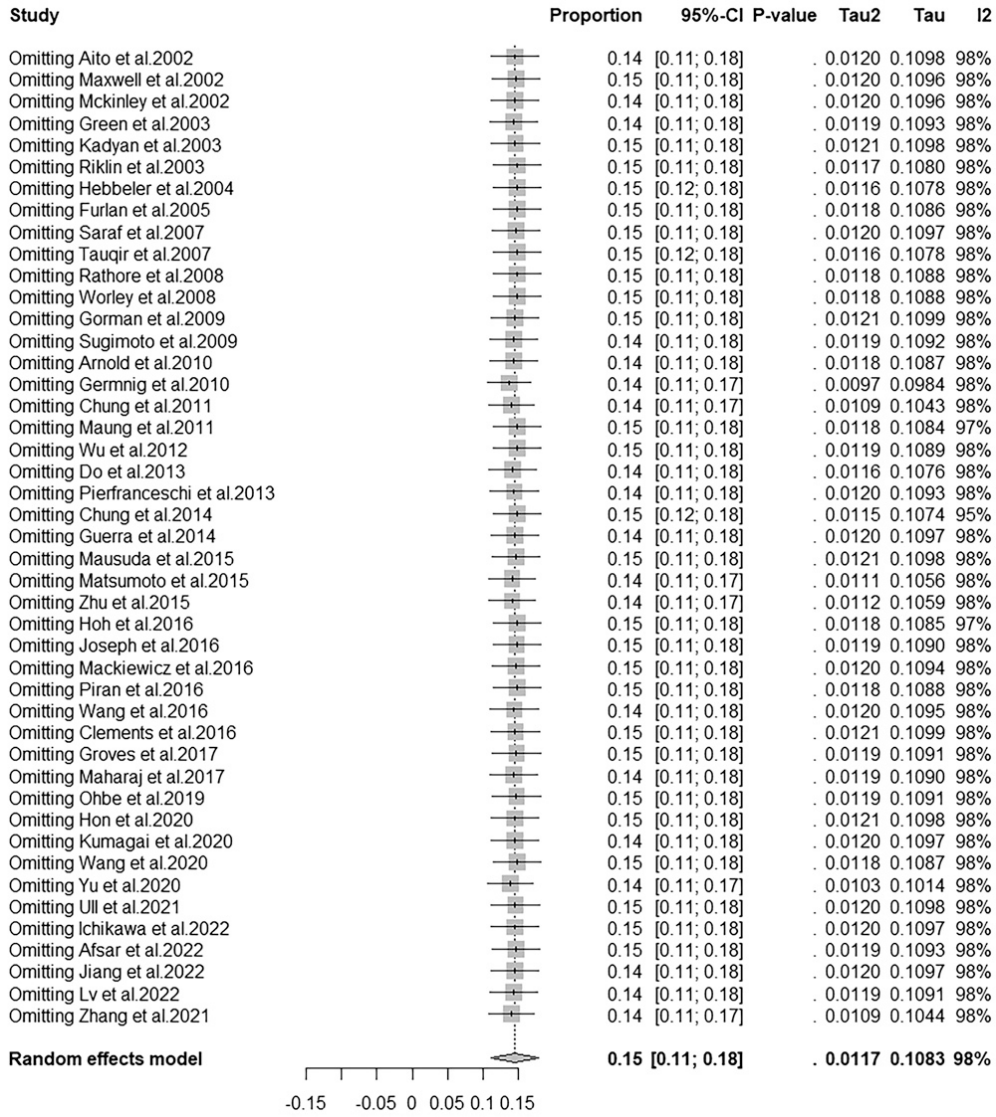
Sensitive analysis.

We performed a sensitivity analysis to explore the impact of each included study on the global prevalence of DVT in patients with SCI. It was proved that no single pooled study would affect the combined results, and our meta-analysis was relatively stable, as shown in Figure 4.

## Discussion

### Summary of results

To the best of our knowledge, this is the first systematic review and meta-analysis to comprehensively analyze the global prevalence of DVT after SCI and the relationship between prevalence and the possible variables (different regions, gender, nerve defect degree, sample source, and thromboprophylaxis). Our analysis revealed that the overall prevalence of DVT in patients with SCI was 14.53% and seemed to be more pronounced in male adults older than 18 during the acute phase. However, the current study does not offer a comparison with non-SCI groups or individuals with other comorbidities, and this will be further validated in future studies. Compared with the patients with SCI from hospitals, those from rehabilitation institutions had a lower prevalence of DVT. Evidence suggests that undergoing rehabilitation can significantly decrease the likelihood of DVT after SCI. For instance, hydrotherapy acts as a vital rehabilitation tool by boosting muscle strength, joint movement, and cardiovascular function, thereby promoting recovery, and decreasing the risk of DVT from SCI.^63^ Those patients with SCI with thoracic-level lesions and ASIA grade A had the highest prevalence of DVT. Patients receiving both mechanical and pharmacological prophylaxis exhibited a reduced incidence compared with those who underwent purely mechanical prophylaxis. In addition, the prevalence of DVT in patients with SCI was similar in Europe, Asia, and South America, fluctuating between 16.86% and 17.02%, while Oceania had the highest prevalence of 18%. Furthermore, including studies with diverse sample sizes (ranging from 24 to 47,916) might bias pooled estimates; for example, larger studies may disproportionately influence the results. In addition, surgical interventions likely influence the risk of DVT. These factors may affect the reliability of the results; however, they were not further explored during the analyses in this study. Ultimately, given the considerable variability among the studies incorporated, our conclusions warrant careful consideration.

### Comparisons with studies

The prevalence of DVT varies with race and region. Previous studies have generally assumed that people in Asia have lower rates of venous thrombosis than those in Western countries.^34,66,67^ However, in our study, it seemed that the incidence gap was narrowing. The trend may be attributed to factors such as the underdiagnosis and underreporting observed in earlier studies, advancements in diagnostic methods in later years, and a heightened awareness among clinicians regarding the issue of VTE within the Asian population.^68^ In addition, with the development of industrialization in Asian countries, lifestyle changes, including diet, had resulted in higher consumption of high-fat diets and urban lifestyles. Risk factors for developing DVT, such as obesity and heart disease, were increasing in Asian countries.

In our analysis, the rate of DVT in patients with SCI was the highest at the thoracic level, followed by the lumbar and cervical level, which may be related to the high incidence of traumatic injuries of the thoracic and lumbar spine in trauma patients.^69–73^ Cervical SCI is usually associated with severe paralysis and a range of complications and has a higher mortality rate. Moreover, patients with SCI with ASIA grade A (complete lesion) had the highest prevalence rate of DVT, presenting a decreasing trend from grade C to grade D (incomplete lesion). Anderson et al. reported that immobility was a risk factor for VTE and persons classified as ASIA grade D were more likely to be ambulating soon after injury.^74,75^ In accordance with us, Watson et al.^76,77^ also indicated a higher incidence of thromboembolism in the complete lesion and the thoracic lesion.

Our combined results suggested that patients with SCI from rehabilitation institutions had a lower prevalence of DVT than those from hospitals. On one hand, this phenomenon may be attributed to the physical therapy and the guidance offered to patients and caregivers by rehabilitation institutions^39^; on the other hand, the disparity in disease stage and severity among patients from hospitals compared with those in rehabilitation institutions could also account for the lower incidence of DVT observed in the latter settings. It was emphasized that differences in the incidence of SCI between hospitals and rehabilitation centers may result from better care practices or differences in patient severity. In subgroup analysis by gender, the prevalence of DVT in males was higher than in females. It might be demonstrated that anti-inflammatory effects, increased blood flow in post-traumatic tissue, upregulation of antiapoptotic Bcl-2, and decreased calcium inflow after trauma, are some of the possible mechanisms of estrogen’s influence on recovery after SCI.^78,79^ STRING functional network analysis of estrogen-regulated proteins after SCI showed that estrogen simultaneously upregulates known neuroprotective pathways, such as HIF-1, and downregulates proinflammatory pathways, including IL-17. These findings highlight the strong therapeutic potential of estrogens and estrogenic compounds after SCI.^80^

Currently, the approaches to VTE prophylaxis encompass mechanical interventions such as pressure-graded elastic stockings, intermittent pneumatic compression, venous foot pumps, and neuromuscular electrical stimulation. In addition, pharmacological prophylaxis includes oral anticoagulants, low-dose unfractionated heparin (LDUH), and low-molecular-weight heparin (LMWH). Surgical options also exist, notably the implantation of inferior vena cava filters, which serve as a preventive measure against PE.^16,81,82^ Subgroup analysis by thromboprophylaxis indicated that the pooled prevalence of DVT in patients with SCI adopting mechanical and pharmacological prophylaxis was lower than mechanical alone. However, Chen et al.’s meta-analysis showed that neither LDUH nor LMWH had a thromboprophylaxis effect compared with placebo or untreated patients with acute SCI.^10^ An evidence-based analysis demonstrated that LMWH is superior to unfractionated heparin in preventing DVT within the SCI population, exhibiting a reduced incidence of bleeding complications.^83^

Many previous studies reported that older age is an independent risk factor for VTE.^22,37,38,45^ The studies we included were not able to stratify by age in detail due to the inconsistent age of the data given. An evidence-based analysis demonstrated that LMWH is superior to unfractionated heparin in preventing DVT within the SCI population, exhibiting a reduced incidence of bleeding complications.^84^

### Implication for clinical practice and health policy

Clinically, we should carefully observe for DVT symptoms, such as swelling, pain, changes in the skin color, or cramping in the calf, and the PE symptoms, such as tachypnea, chest pain, tachycardia, and hypotension.^85^ However, it is important to note that some patients would not exhibit typical DVT symptoms, which may lead to delayed onset of the disease. Therefore, special attention should be given to adult male patients with acute SCI at the thoracic level with AISA grade A. With contraindications excluded, we should use mechanical combined with pharmacological prophylaxis early after SCI.

### Strengths and limitations

The strengths of this analysis include a comprehensive and systematic search strategy, along with the global representativeness of the included studies. We performed an in-depth subgroup analysis and explored the influence of various factors on the prevalence of DVT in patients with SCI from the demographic characteristics, regional distribution, the nerve defect degree, and different thromboprophylaxis. There are several limitations in our meta-analysis. First, our population size ranged from 24 to 47,916, and different diagnostic methods, such as venography and color ultrasonic, may induce bias. Second, most studies lack detailed age stratification, limiting further analysis. Third, the databases included may introduce bias in the studies based on regional representation. In addition, diagnostic methods may vary significantly in sensitivity and specificity and influence pooled prevalence estimates. More research is needed in the future to validate this result. Finally, the impact of surgery or fracture following SCI on the prevalence of venous thrombosis was excluded.

## Conclusions

This study revealed the global prevalence of DVT in patients with SCI in terms of gender, sample source, population distribution, nerve defect degree, and thromboprophylaxis. The estimated overall pooled prevalence of DVT after SCI was 14.53%. The highest prevalence rate was found in Europe, among adult male patients with acute SCI at the thoracic level with AISA grade A. Given the significant variability of the studies included, our results should be interpreted with caution. In the future, screening for venous thrombosis in patients with SCI should be increased to identify asymptomatic patients early and to further explore the best prevention or treatment methods to improve the prognosis.

## Transparency, Rigor, and Reproducibility Summary

The systematic review of DVT in patients with SCI exemplified transparency, rigor, and repeatability throughout its methodology and reporting. The comprehensive search across five reputable databases, from 2000 to March 2023, ensured a thorough examination of relevant literature on DVT prevalence among patients with SCI, with clear inclusion criteria for observational descriptive studies. Data extraction encompassed varied aspects such as author details, demographic characteristics, and sample sources, underscoring the meticulous approach to data collection. The assessment of bias using the Joanna Briggs Institute Critical Appraisal Instrument and the utilization of random-effects models for data synthesis highlighted the rigorous analytical framework adopted in the review. The estimation of the overall pooled prevalence of DVT, stratified by patient characteristics and health care settings, facilitated a nuanced understanding of the issue’s epidemiology. Factors influencing DVT prevalence in patients with SCI, including regional distribution, demographic features, nerve defect severity, lesion levels, and thromboprophylaxis, were comprehensively explored, adding depth to the analysis. While the findings serve as a valuable reference for future epidemiological studies, the acknowledgment of high heterogeneity among included studies emphasized the necessity for cautious interpretation of results, underscoring the article’s commitment to transparency and the imperative of reproducibility in scientific inquiry.

## Data Availability

All data generated or analyzed during this study are included in this published article [and its supplementary information files].

## Abbreviations Used

DVT: deep vein thrombosis
SCI: spinal cord injury
VTE: venous thromboembolism
NTSCI: nontraumatic spinal cord injury
