# Supplementary material for: Global Prevalence of Deep Vein Thrombosis in Patients with Spinal Cord Injury: A Systematic Review and Meta-Analysis
Source: Neurotrauma Rep. 2025 Jun 12;6(1):491–505. doi: 10.1089/neur.2024.0144 (PMC12235127; doi:10.1089/neur.2024.0144)
Supplement: Supplementary Data [file neur.2024.0144_supplementarydata.docx]

**Supplementary Online Content**

**Supplementary S1.** Search strategy for each database

**Supplementary Figure S1. Prevalence of DVT After SCI by Sample Source**

**Supplementary Figure S2. Prevalence of DVT After SCI by Continent**

**Supplementary Figure S3. Prevalence of DVT After SCI by Gender**

**Supplementary Figure S4. Prevalence of DVT After SCI by Neurological Status**

**Supplementary Figure S5. Prevalence of DVT After SCI by Lesion Level**

**Supplementary Figure S6. Prevalence of DVT After SCI by Thromboprophylaxis**

**Supplementary Figure S7. Prevalence of DVT in acute SCI Patients**

**Supplementary Figure S8. Prevalence of DVT In SCI Over 18 Years Old**

**Supplementary Figure S9. Publication bias**

**Supplementary Table S1: Risk of bias assessment**

**Supplementary eMethods**

**Supplementary S1. Search strategy for each database**

**PUBMED search strategy**

((("Spinal Cord Injuries"[Mesh]) OR ((Spinal Cord Trauma[Title/Abstract]) OR (Cord Trauma, Spinal[Title/Abstract]) OR (Cord Traumas, Spinal[Title/Abstract]) OR (Spinal Cord Traumas[Title/Abstract]) OR (Trauma, Spinal Cord[Title/Abstract]) OR (Traumas, Spinal Cord[Title/Abstract]) OR (Myelopathy, Traumatic[Title/Abstract]) OR (Myelopathies, Traumatic[Title/Abstract]) OR (Traumatic Myelopathies[Title/Abstract]) OR (Traumatic Myelopathy[Title/Abstract]) OR (Injuries, Spinal Cord[Title/Abstract]) OR (Cord Injuries, Spinal[Title/Abstract]) OR (Cord Injury, Spinal[Title/Abstract]) OR (Injury, Spinal Cord[Title/Abstract]) OR (Spinal Cord Injury[Title/Abstract]) OR (Spinal Cord Transection[Title/Abstract]) OR (Cord Transection, Spinal[Title/Abstract]) OR (Cord Transections, Spinal[Title/Abstract]) OR (Spinal Cord Transections[Title/Abstract]) OR (Transection, Spinal Cord[Title/Abstract]) OR (Transections, Spinal Cord[Title/Abstract]) OR (Spinal Cord Laceration[Title/Abstract]) OR (Cord Laceration, Spinal[Title/Abstract]) OR (Cord Lacerations, Spinal[Title/Abstract]) OR (Laceration, Spinal Cord[Title/Abstract]) OR (Lacerations, Spinal Cord[Title/Abstract]) OR (Spinal Cord Lacerations[Title/Abstract]) OR (Post-Traumatic Myelopathy[Title/Abstract]) OR (Myelopathies, Post-Traumatic[Title/Abstract]) OR (spinal fracture[Title/Abstract]))) AND (("Venous Thrombosis"[Mesh]) OR ((Phlebothrombosis[Title/Abstract]) OR (Phlebothromboses[Title/Abstract]) OR (Thrombosis, Venous[Title/Abstract]) OR (Thromboses, Venous[Title/Abstract]) OR (Venous Thromboses[Title/Abstract]) OR (Deep Vein Thrombosis[Title/Abstract]) OR (Deep Vein Thromboses[Title/Abstract]) OR (Thromboses, Deep Vein[Title/Abstract]) OR (Vein Thromboses, Deep[Title/Abstract]) OR (Vein Thrombosis, Deep[Title/Abstract]) OR (Deep-Venous Thrombosis[Title/Abstract]) OR (Deep-Venous Thromboses[Title/Abstract]) OR (Thromboses, Deep-Venous[Title/Abstract]) OR (Thrombosis, Deep-Venous[Title/Abstract]) OR (Deep-Vein Thrombosis[Title/Abstract]) OR (Deep-Vein Thromboses[Title/Abstract]) OR (Thromboses, Deep-Vein[Title/Abstract]) OR (Thrombosis, Deep-Vein[Title/Abstract]) OR (Thrombosis, Deep Vein[Title/Abstract]) OR (Deep Venous Thrombosis[Title/Abstract]) OR (Deep Venous Thromboses[Title/Abstract]) OR (Thromboses, Deep Venous[Title/Abstract]) OR (Thrombosis, Deep Venous[Title/Abstract]) OR (Venous Thromboses, Deep[Title/Abstract]) OR (Venous Thrombosis, Deep[Title/Abstract]) OR (DVT[Title/Abstract])))) AND (((("Epidemiology"[Mesh]) OR ((Social Epidemiology[Title/Abstract]) OR (Epidemiologies, Social[Title/Abstract]) OR (Epidemiology, Social[Title/Abstract]) OR (Social Epidemiologies[Title/Abstract]))) OR (("Incidence"[Mesh]) OR (Incidences[Title/Abstract] OR Incidence Proportion[Title/Abstract] OR Incidence Proportions[Title/Abstract] OR Proportion, Incidence[Title/Abstract] OR Attack Rate[Title/Abstract] OR Attack Rates[Title/Abstract] OR Rate, Attack[Title/Abstract] OR Cumulative Incidence[Title/Abstract] OR Cumulative Incidences[Title/Abstract] OR Incidence, Cumulative[Title/Abstract] OR Incidence Rate[Title/Abstract] OR Incidence Rates[Title/Abstract] OR Rate, Incidence[Title/Abstract]))) OR (("Prevalence"[Mesh]) OR (Prevalences[Title/Abstract] OR Period Prevalence[Title/Abstract] OR Period Prevalences[Title/Abstract] OR Prevalence, Period[Title/Abstract] OR Point Prevalence[Title/Abstract] OR Point Prevalences[Title/Abstract] OR Prevalence, Point[Title/Abstract])))

**EMBASE search strategy**

#1. 'spinal cord injury'/exp

#2. 'spinal cord injuries':ab,ti OR 'spinal cord

trauma':ab,ti OR 'cord trauma, spinal':ab,ti OR

'cord traumas, spinal':ab,ti OR 'spinal cord

traumas':ab,ti OR 'trauma, spinal cord':ab,ti OR

'traumas, spinal cord':ab,ti OR 'myelopathy,

traumatic':ab,ti OR 'myelopathies,

traumatic':ab,ti OR 'traumatic

myelopathies':ab,ti OR 'traumatic

myelopathy':ab,ti OR 'injuries, spinal

cord':ab,ti OR 'cord injuries, spinal':ab,ti OR

'cord injury, spinal':ab,ti OR 'injury, spinal

cord':ab,ti OR 'spinal cord injury':ab,ti OR

'spinal cord transection':ab,ti OR 'cord

transection, spinal':ab,ti OR 'cord transections,

spinal':ab,ti OR 'spinal cord transections':ab,ti

OR 'transection, spinal cord':ab,ti OR

'transections, spinal cord':ab,ti OR 'spinal cord

laceration':ab,ti OR 'cord laceration,

spinal':ab,ti OR 'cord lacerations, spinal' OR

'laceration, spinal cord' OR 'lacerations, spinal

cord' OR 'spinal cord lacerations' OR

'post-traumatic myelopathy' OR 'myelopathies,

post-traumatic' OR 'myelopathy, post-traumatic'

OR 'post traumatic myelopathy' OR 'post-traumatic

myelopathies':ab,ti OR 'spinal cord

contusion':ab,ti OR 'contusion, spinal

cord':ab,ti OR 'contusions, spinal cord':ab,ti OR

'cord contusion, spinal':ab,ti OR 'cord

contusions, spinal':ab,ti OR 'spinal cord

contusions':ab,ti OR 'sci':ab,ti OR 'spinal

injury':ab,ti OR 'spinal fracture':ab,ti

#3. #1 OR #2

#4. 'vein thrombosis'/exp

#5. 'venous thrombosis':ab,ti OR

'phlebothrombosis':ab,ti OR

'phlebothromboses':ab,ti OR 'thrombosis,

venous':ab,ti OR 'thromboses, venous':ab,ti OR

'venous thromboses':ab,ti OR 'deep vein

thrombosis':ab,ti OR 'deep vein thromboses':ab,ti

OR 'thromboses, deep vein':ab,ti OR 'vein

thromboses, deep':ab,ti OR 'vein thrombosis,

deep':ab,ti OR 'deep-venous thrombosis':ab,ti OR

'deep-venous thromboses':ab,ti OR 'thromboses,

deep-venous':ab,ti OR 'thrombosis,

deep-venous':ab,ti OR 'deep-vein

thrombosis':ab,ti OR 'deep-vein thromboses':ab,ti

OR 'thromboses, deep-vein':ab,ti OR 'thrombosis,

deep-vein':ab,ti OR 'thrombosis, deep vein':ab,ti

OR 'deep venous thrombosis':ab,ti OR 'deep venous

thromboses':ab,ti OR 'thromboses, deep

venous':ab,ti OR 'thrombosis, deep venous':ab,ti

OR 'venous thromboses, deep':ab,ti OR 'venous

thrombosis, deep':ab,ti OR 'dvt':ab,ti OR

'phlebo-thrombosis':ab,ti OR 'vena

thrombosis':ab,ti OR 'venothrombosis':ab,ti OR

'venothrombotic event':ab,ti

#6. #4 OR #5

#7. 'epidemiology'/exp

#8. 'social epidemiology':ab,ti OR 'epidemiologies,

social':ab,ti OR 'epidemiology, social':ab,ti OR

'social epidemiologies':ab,ti

#9. #7 OR #8

#10. 'incidence'/exp

#11. 'incidences':ab,ti OR 'incidence

proportion':ab,ti OR 'incidence

proportions':ab,ti OR 'proportion,

incidence':ab,ti OR 'attack rate':ab,ti OR

'attack rates':ab,ti OR 'rate, attack':ab,ti OR

'cumulative incidence':ab,ti OR 'cumulative

incidences':ab,ti OR 'incidence,

cumulative':ab,ti OR 'incidence rate':ab,ti OR

'incidence rates':ab,ti OR 'rate,

incidence':ab,ti

#12. #10 OR #11

#13. 'prevalence'/exp

#14. 'prevalences':ab,ti OR 'period prevalence':ab,ti

OR 'period prevalences':ab,ti OR 'prevalence,

period':ab,ti OR 'point prevalence':ab,ti OR

'point prevalences':ab,ti OR 'prevalence,

point':ab,ti

#15. #13 OR #14

#16. #9 OR #12 OR #15

#17. #3 AND #6 AND #16

**COCHRAIN search strategy**

#1 MeSH descriptor: [Spinal Cord Injuries] explode all trees

#2 (Spinal Cord Trauma or Cord Trauma, Spinal or Cord Traumas, Spinal or Spinal Cord Traumas or Trauma, Spinal Cord or Traumas, Spinal Cord or Myelopathy, Traumatic or Myelopathies, Traumatic or Traumatic Myelopathies or Traumatic Myelopathy or Injuries, Spinal Cord or Cord Injuries, Spinal or Cord Injury, Spinal or Injury, Spinal Cord or Spinal Cord Injury or Spinal Cord Transection or Cord Transection, Spinal or Cord Transections, Spinal or Spinal Cord Transections or Transection, Spinal Cord or Transections, Spinal Cord or Spinal Cord Laceration or Cord Laceration, Spinal or Cord Lacerations, Spinal or Laceration, Spinal Cord or Lacerations, Spinal Cord or Spinal Cord Lacerations or Post-Traumatic Myelopathy or Myelopathies, Post-Traumatic or Myelopathy, Post-Traumatic or Post Traumatic Myelopathy or Post-Traumatic Myelopathies or Spinal Cord Contusion or Contusion, Spinal Cord or Contusions, Spinal Cord or Cord Contusion, Spinal or Cord Contusions, Spinal or Spinal Cord Contusions or SCI or spinal injury or spinal fracture):ti,ab,kw (Word variations have been searched)

#3 #1 or #2

#4 MeSH descriptor: [Venous Thrombosis] explode all trees

#5 (Phlebothrombosis or Phlebothromboses or Thrombosis, Venous or Thromboses, Venous or Venous Thromboses or Deep Vein Thrombosis or Deep Vein Thromboses or Thromboses, Deep Vein or Vein Thromboses, Deep or Vein Thrombosis, Deep or Deep-Venous Thrombosis or Deep-Venous Thromboses or Thromboses, Deep-Venous or Thrombosis, Deep-Venous or Deep-Vein Thrombosis or Deep-Vein Thromboses or Thromboses, Deep-Vein or Thrombosis, Deep-Vein or Thrombosis, Deep Vein or Deep Venous Thrombosis or Deep Venous Thromboses or Thromboses, Deep Venous or Thrombosis, Deep Venous or Venous Thromboses, Deep or Venous Thrombosis, Deep or DVT or phlebo-thrombosis or vena thrombosis or venothrombosis or venothrombotic event):ti,ab,kw (Word variations have been searched)

#6 #4 or #5

#7 MeSH descriptor: [Epidemiology] explode all trees

#8 (Social Epidemiology or Epidemiologies, Social or Epidemiology, Social or Social Epidemiologies):ti,ab,kw (Word variations have been searched)

#9 #7 or #8

#10 MeSH descriptor: [Incidence] explode all trees

#11 (Incidences or Incidence Proportion or Incidence Proportions or Proportion, Incidence or Attack Rate or Attack Rates or Rate, Attack or Cumulative Incidence or Cumulative Incidences or Incidence, Cumulative or Incidence Rate or Incidence Rates or Rate, Incidence):ti,ab,kw (Word variations have been searched)

#12 #10 or #11

#13 MeSH descriptor: [Prevalence] explode all trees

#14 (Prevalences or Period Prevalence or Period Prevalences or Prevalence, Period or Point Prevalence or Point Prevalences or Prevalence, Point):ti,ab,kw (Word variations have been searched)

#15 #13 or #14

#16 #9 or #12 or #15

#17 #3 and #6 and #16

**SCOPUS search strategy**

TITLE-ABS-KEY ( "Spinal Cord Injuries" OR "Spinal Cord Trauma" OR "Cord Trauma, Spinal" OR "Cord Traumas, Spinal" OR "Spinal Cord Traumas" OR "Trauma, Spinal Cord" OR "Traumas, Spinal Cord" OR "Myelopathy, Traumatic" OR "Myelopathies, Traumatic" OR "Traumatic Myelopathies" OR "Traumatic Myelopathy" OR "Injuries, Spinal Cord" OR "Cord Injuries, Spinal" OR "Cord Injury, Spinal" OR "Injury, Spinal Cord" OR "Spinal Cord Injury" OR "Spinal Cord Transection" OR "Cord Transection, Spinal" OR "Cord Transections, Spinal" OR "Spinal Cord Transections" OR "Transection, Spinal Cord" OR "Transections, Spinal Cord" OR "Spinal Cord Laceration" OR "Cord Laceration, Spinal" OR "Cord Lacerations, Spinal" OR "Laceration, Spinal Cord" OR "Lacerations, Spinal Cord" OR "Spinal Cord Lacerations" OR "Post-Traumatic Myelopathy" OR "Myelopathies, Post-Traumatic" OR "Myelopathy, Post-Traumatic" OR "Post Traumatic Myelopathy" OR "Post-Traumatic Myelopathies" OR "Spinal Cord Contusion" OR "Contusion, Spinal Cord" OR "Contusions, Spinal Cord" OR "Cord Contusion, Spinal" OR "Cord Contusions, Spinal" OR "Spinal Cord Contusions" OR "SCI" OR "spinal injury" OR "spinal fracture" )

TITLE-ABS-KEY ( "Venous Thrombosis" OR "Phlebothrombosis" OR "Phlebothromboses" OR "Thrombosis, Venous" OR "Thromboses, Venous" OR "Venous Thromboses" OR "Deep Vein Thrombosis" OR "Deep Vein Thromboses" OR "Thromboses, Deep Vein" OR "Vein Thromboses, Deep" OR "Vein Thrombosis, Deep" OR "Deep-Venous Thrombosis" OR "Deep-Venous Thromboses" OR "Thromboses, Deep-Venous" OR "Thrombosis, Deep-Venous" OR "Deep-Vein Thrombosis" OR "Deep-Vein Thromboses" OR " Thromboses, Deep-Vein" OR " Thrombosis, Deep-Vein" OR "Thrombosis, Deep Vein" OR "Deep Venous Thrombosis" OR "Deep Venous Thromboses" OR "Thromboses, Deep Venous" OR "Thrombosis, Deep Venous" OR "Venous Thromboses, Deep" OR "Venous Thrombosis, Deep" OR "DVT" OR "phlebo-thrombosis" OR "vena thrombosis" OR "venothrombosis" OR "venothrombotic event" )

TITLE-ABS-KEY ( "Epidemiology" OR "Social Epidemiology" OR "Epidemiologies, Social" OR "Epidemiology, Social" OR "Social Epidemiologies" OR "Incidence" OR "Incidences" OR "Incidence Proportion" OR "Incidence Proportions" OR "Proportion, Incidence" OR "Attack Rate" OR "Attack Rates" OR "Rate, Attack" OR "Cumulative Incidence" OR "Cumulative Incidences" OR "Incidence, Cumulative" OR "Incidence Rate" OR "Incidence Rates" OR "Rate, Incidence" OR "Prevalence" OR "Prevalences" OR "Period Prevalence" OR "Period Prevalences" OR "Prevalence, Period" OR "Point Prevalence" OR "Point Prevalences" OR "Prevalence, Point" )

( TITLE-ABS-KEY ( "Spinal Cord Injuries" OR "Spinal Cord Trauma" OR "Cord Trauma, Spinal" OR "Cord Traumas, Spinal" OR "Spinal Cord Traumas" OR "Trauma, Spinal Cord" OR "Traumas, Spinal Cord" OR "Myelopathy, Traumatic" OR "Myelopathies, Traumatic" OR "Traumatic Myelopathies" OR "Traumatic Myelopathy" OR "Injuries, Spinal Cord" OR "Cord Injuries, Spinal" OR "Cord Injury, Spinal" OR "Injury, Spinal Cord" OR "Spinal Cord Injury" OR "Spinal Cord Transection" OR "Cord Transection, Spinal" OR "Cord Transections, Spinal" OR "Spinal Cord Transections" OR "Transection, Spinal Cord" OR "Transections, Spinal Cord" OR "Spinal Cord Laceration" OR "Cord Laceration, Spinal" OR "Cord Lacerations, Spinal" OR "Laceration, Spinal Cord" OR "Lacerations, Spinal Cord" OR "Spinal Cord Lacerations" OR "Post-Traumatic Myelopathy" OR "Myelopathies, Post-Traumatic" OR "Myelopathy, Post-Traumatic" OR "Post Traumatic Myelopathy" OR "Post-Traumatic Myelopathies" OR "Spinal Cord Contusion" OR "Contusion, Spinal Cord" OR "Contusions, Spinal Cord" OR "Cord Contusion, Spinal" OR "Cord Contusions, Spinal" OR "Spinal Cord Contusions" OR "SCI" OR "spinal injury" OR "spinal fracture" ) ) AND ( TITLE-ABS-KEY ( "Venous Thrombosis" OR "Phlebothrombosis" OR "Phlebothromboses" OR "Thrombosis, Venous" OR "Thromboses, Venous" OR "Venous Thromboses" OR "Deep Vein Thrombosis" OR "Deep Vein Thromboses" OR "Thromboses, Deep Vein" OR "Vein Thromboses, Deep" OR "Vein Thrombosis, Deep" OR "Deep-Venous Thrombosis" OR "Deep-Venous Thromboses" OR "Thromboses, Deep-Venous" OR "Thrombosis, Deep-Venous" OR "Deep-Vein Thrombosis" OR "Deep-Vein Thromboses" OR " Thromboses, Deep-Vein" OR " Thrombosis, Deep-Vein" OR "Thrombosis, Deep Vein" OR "Deep Venous Thrombosis" OR "Deep Venous Thromboses" OR "Thromboses, Deep Venous" OR "Thrombosis, Deep Venous" OR "Venous Thromboses, Deep" OR "Venous Thrombosis, Deep" OR "DVT" OR "phlebo-thrombosis" OR "vena thrombosis" OR "venothrombosis" OR "venothrombotic event" ) ) AND ( TITLE-ABS-KEY ( "Epidemiology" OR "Social Epidemiology" OR "Epidemiologies, Social" OR "Epidemiology, Social" OR "Social Epidemiologies" OR "Incidence" OR "Incidences" OR "Incidence Proportion" OR "Incidence Proportions" OR "Proportion, Incidence" OR "Attack Rate" OR "Attack Rates" OR "Rate, Attack" OR "Cumulative Incidence" OR "Cumulative Incidences" OR "Incidence, Cumulative" OR "Incidence Rate" OR "Incidence Rates" OR "Rate, Incidence" OR "Prevalence" OR "Prevalences" OR "Period Prevalence" OR "Period Prevalences" OR "Prevalence, Period" OR "Point Prevalence" OR "Point Prevalences" OR "Prevalence, Point" ) )

**WEB OF SCIENCE search strategy**

1: TS=(Spinal Cord Injuries or Spinal Cord Trauma or Cord Trauma, Spinal or Cord Traumas, Spinal or Spinal Cord Traumas or Trauma, Spinal Cord or Traumas, Spinal Cord or Myelopathy, Traumatic or Myelopathies, Traumatic or Traumatic Myelopathies or Traumatic Myelopathy or Injuries, Spinal Cord or Cord Injuries, Spinal or Cord Injury, Spinal or Injury, Spinal Cord or Spinal Cord Injury or Spinal Cord Transection or Cord Transection, Spinal or Cord Transections, Spinal or Spinal Cord Transections or Transection, Spinal Cord or Transections, Spinal Cord or Spinal Cord Laceration or Cord Laceration, Spinal or Cord Lacerations, Spinal or Laceration, Spinal Cord or Lacerations, Spinal Cord or Spinal Cord Lacerations or Post-Traumatic Myelopathy or Myelopathies, Post-Traumatic or Myelopathy, Post-Traumatic or Post Traumatic Myelopathy or Post-Traumatic Myelopathies or Spinal Cord Contusion or Contusion, Spinal Cord or Contusions, Spinal Cord or Cord Contusion, Spinal or Cord Contusions, Spinal or Spinal Cord Contusions or SCI or spinal injury or spinal fracture)

2: TS=(Phlebothrombosis or Phlebothromboses or Thrombosis, Venous or Thromboses, Venous or Venous Thromboses or Deep Vein Thrombosis or Deep Vein Thromboses or Thromboses, Deep Vein or Vein Thromboses, Deep or Vein Thrombosis, Deep or Deep-Venous Thrombosis or Deep-Venous Thromboses or Thromboses, Deep-Venous or Thrombosis, Deep-Venous or Deep-Vein Thrombosis or Deep-Vein Thromboses or Thromboses, Deep-Vein or Thrombosis, Deep-Vein or Thrombosis, Deep Vein or Deep Venous Thrombosis or Deep Venous Thromboses or Thromboses, Deep Venous or Thrombosis, Deep Venous or Venous Thromboses, Deep or Venous Thrombosis, Deep or DVT or phlebo-thrombosis or vena thrombosis or venothrombosis or venothrombotic event)

3: TS=(Epidemiology or Social Epidemiology or Epidemiologies, Social or Epidemiology, Social or Social Epidemiologies or Incidence or Incidences or Incidence Proportion or Incidence Proportions or Proportion, Incidence or Attack Rate or Attack Rates or Rate, Attack or Cumulative Incidence or Cumulative Incidences or Incidence, Cumulative or Incidence Rate or Incidence Rates or Rate, Incidence or Prevalence or Prevalences or Period Prevalence or Period Prevalences or Prevalence, Period or Point Prevalence or Point Prevalences or Prevalence, Point)

4: #1 AND #2 AND #3

**Supplementary Figure S1. Prevalence of DVT After SCI by Sample Source**


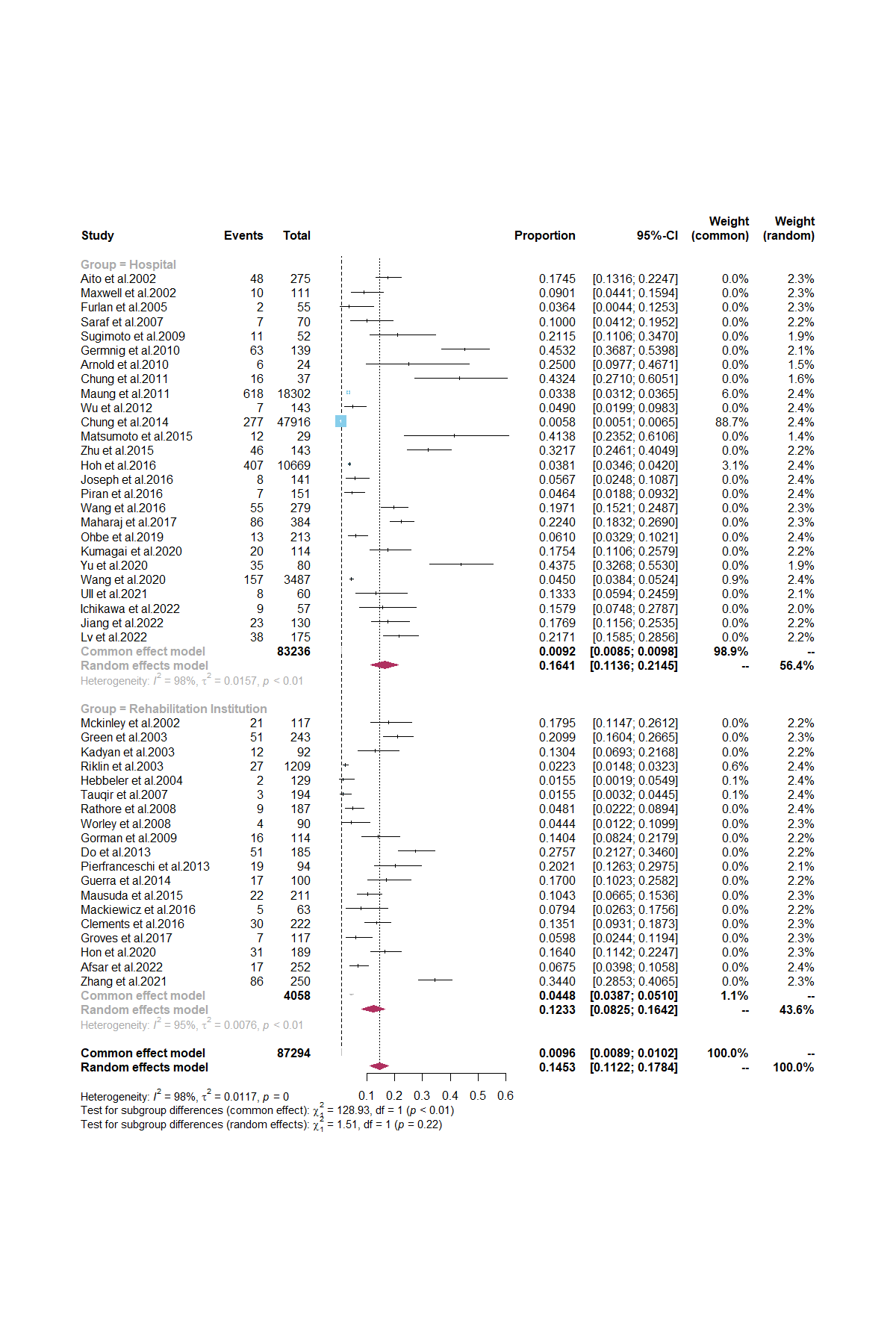


**Supplementary Figure S2. Prevalence of DVT After SCI by Continent**

**
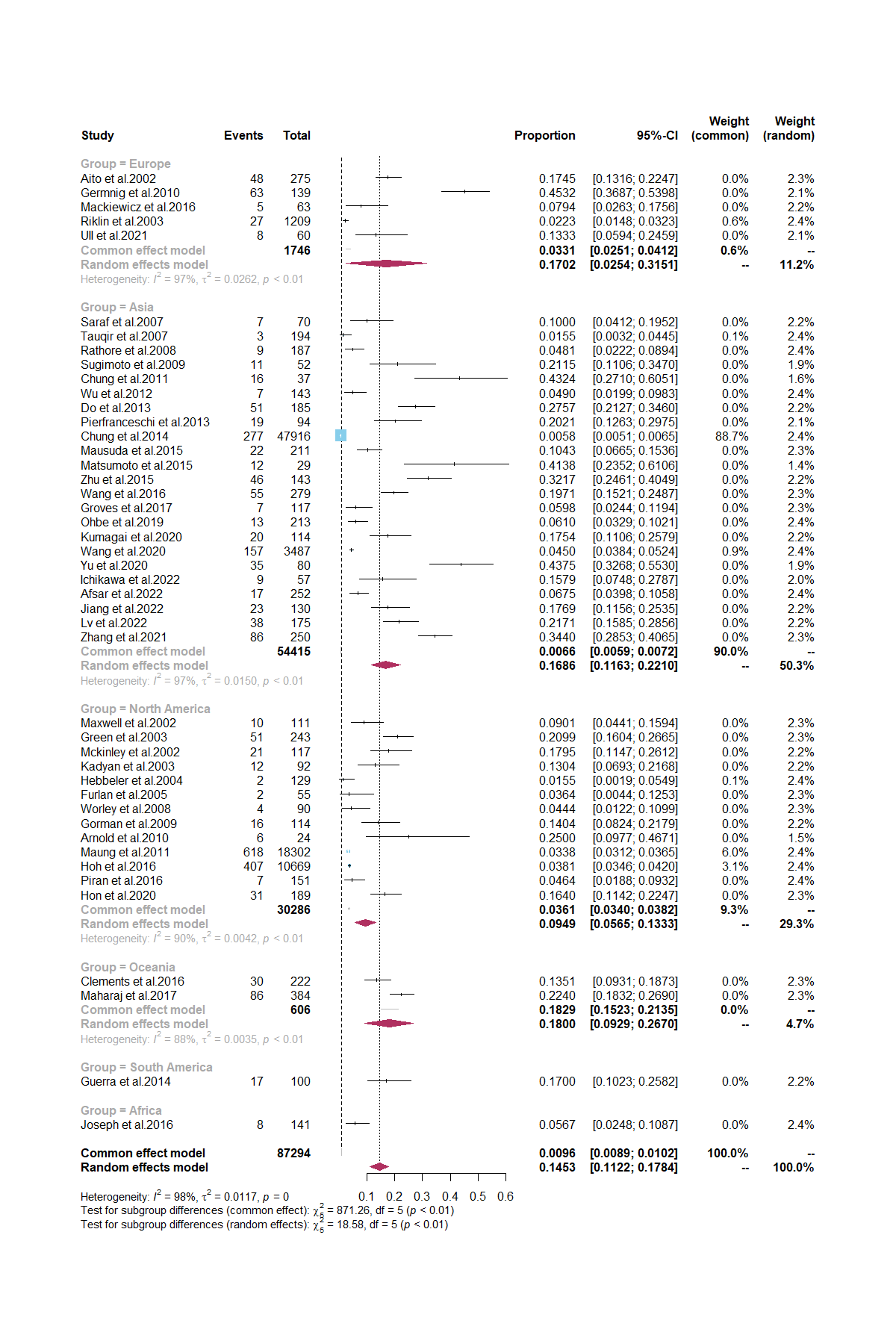
**

**Supplementary Figure S3. Prevalence of DVT After SCI by Gender**


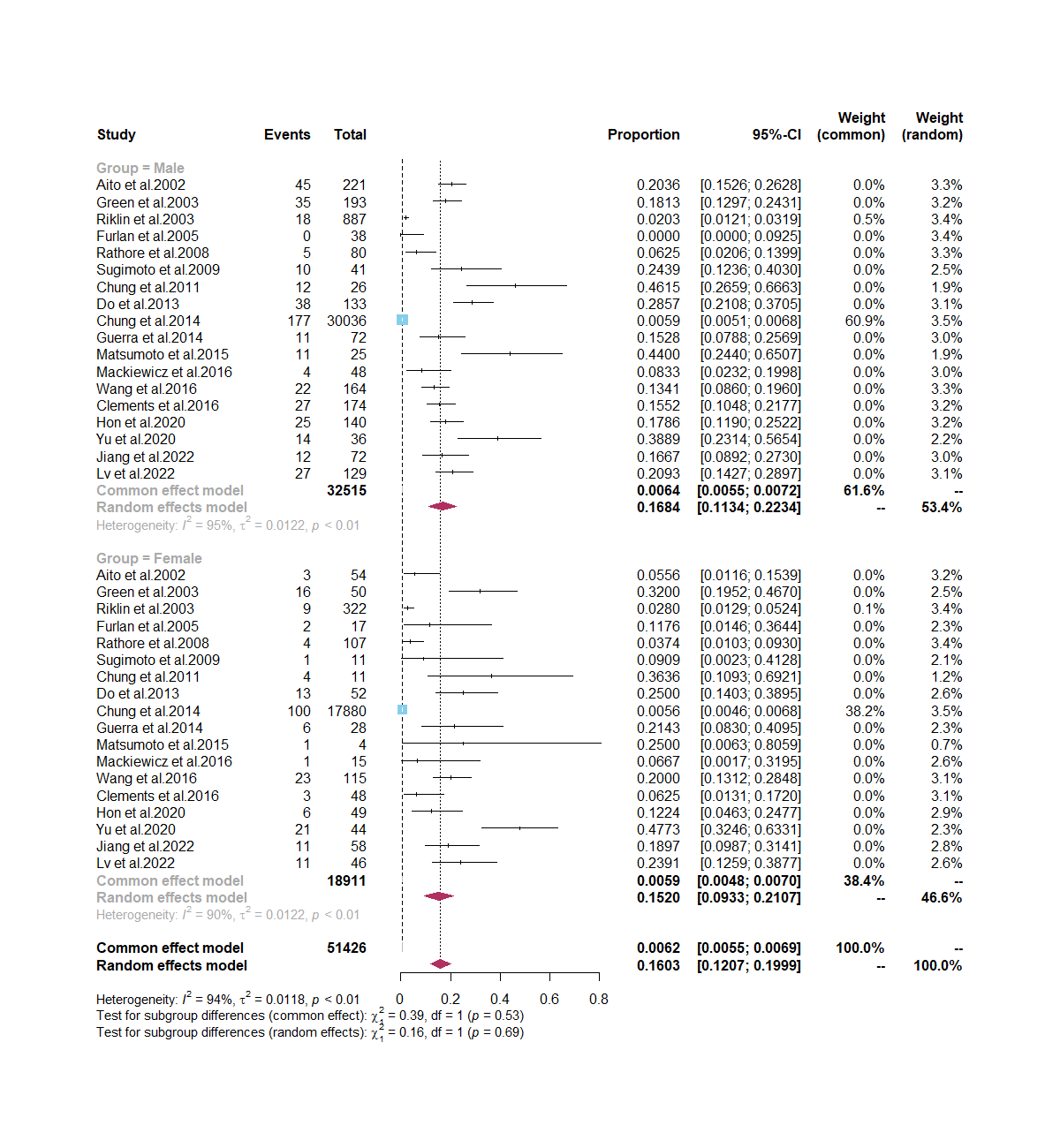


**Supplementary Figure S4. Prevalence of DVT After SCI by Neurological Status**

**
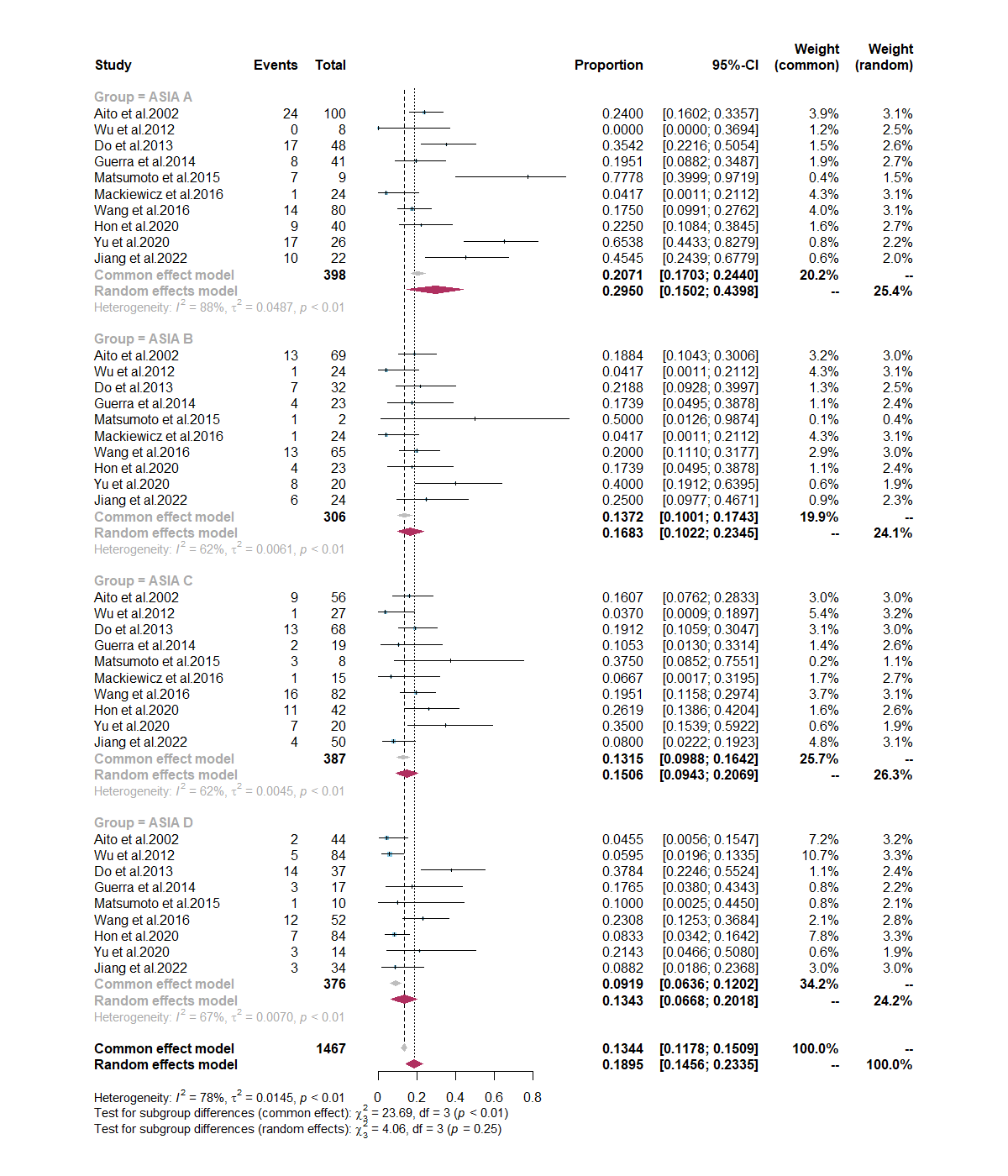
**

**Supplementary Figure S5. Prevalence of DVT After SCI by Lesion Level**

**
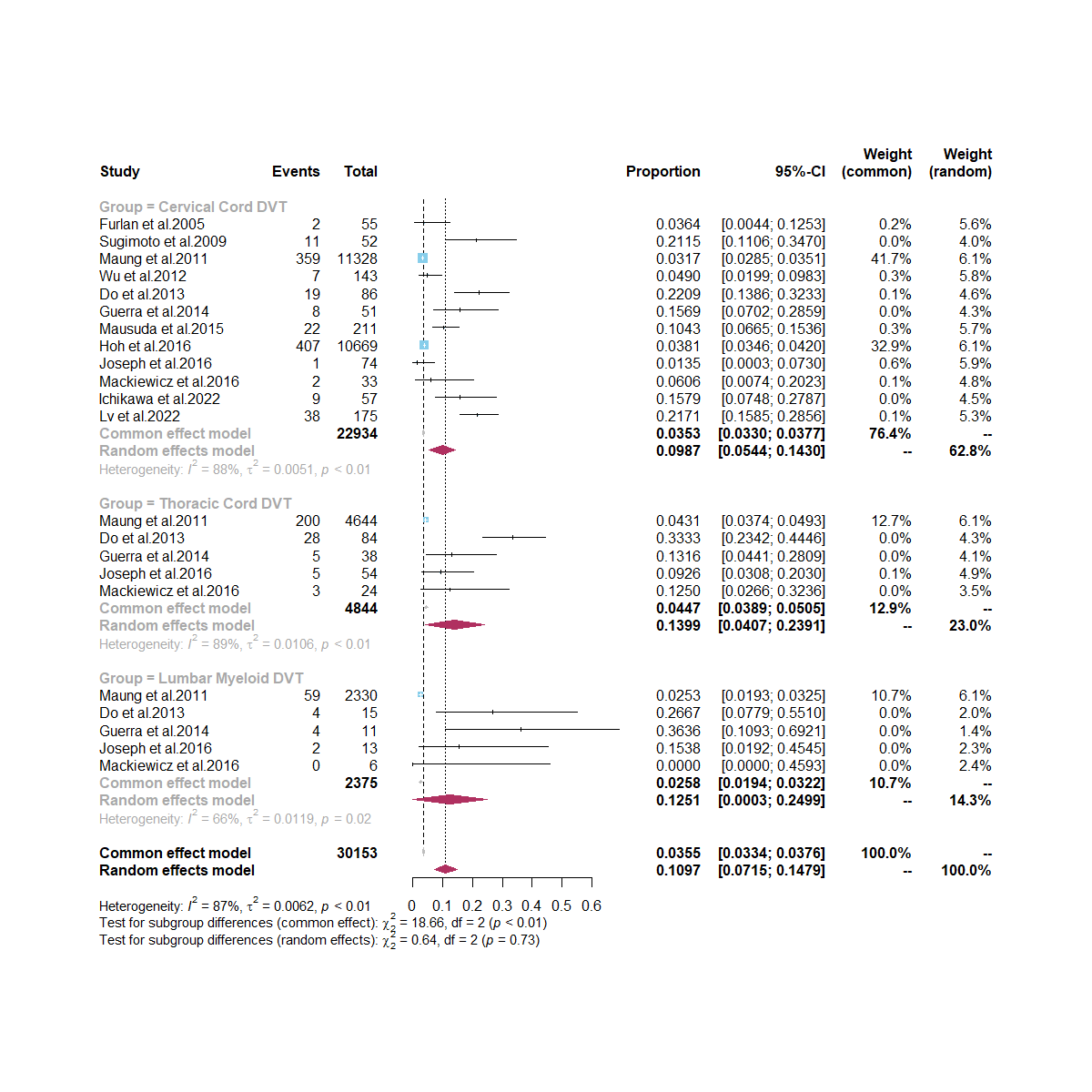
**

**Supplementary Figure S6. Prevalence of DVT After SCI by** **Thromboprophylaxis**

**
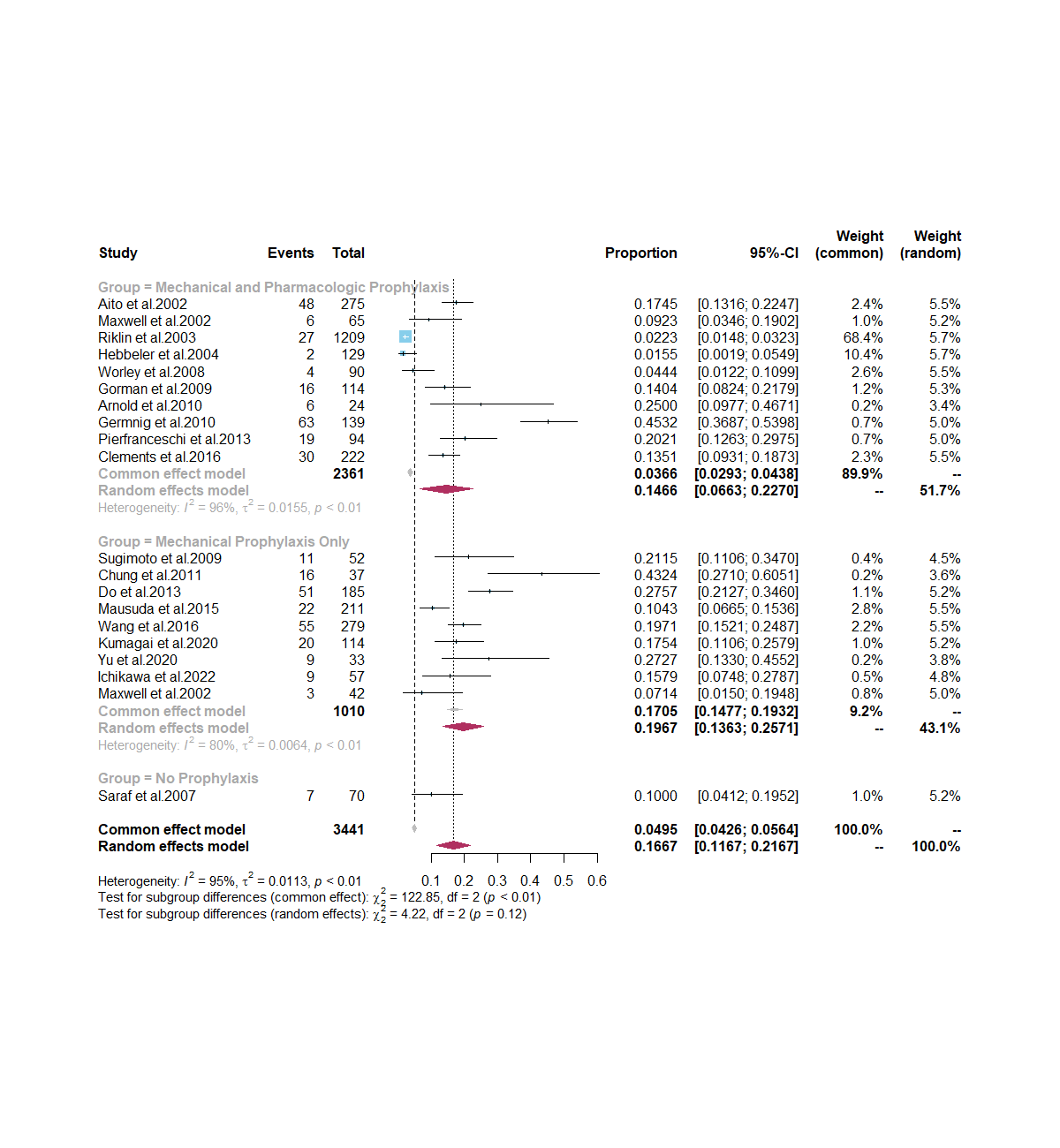
**

**Supplementary Figure S7. Prevalence of DVT in acute SCI Patients**

**
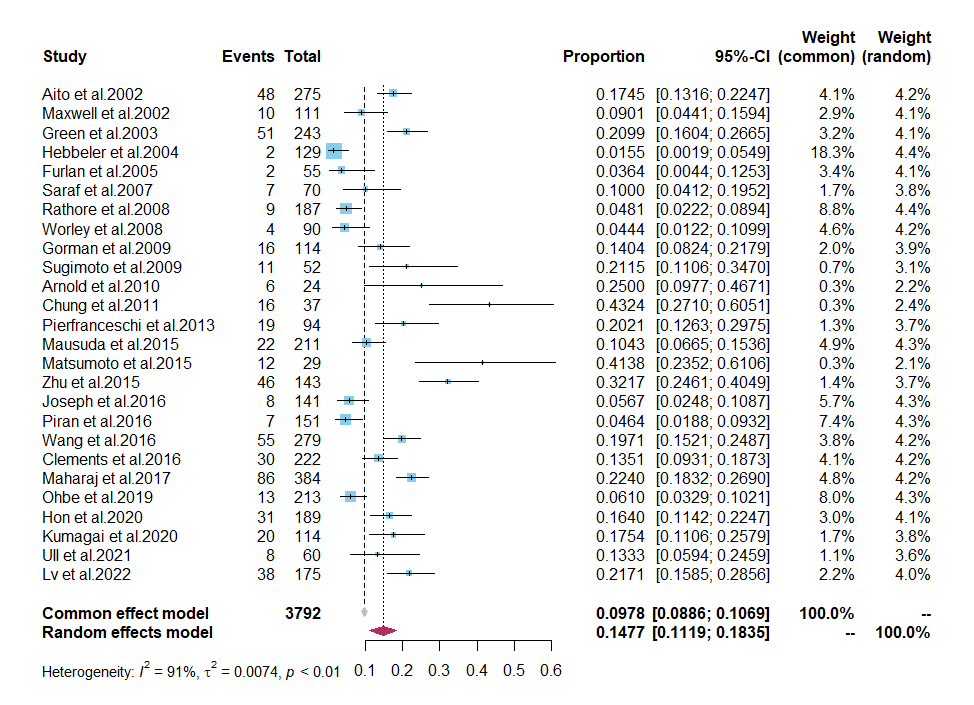
**

**Supplementary Figure S8. Prevalence of DVT In SCI Over 18 Years Old**

**
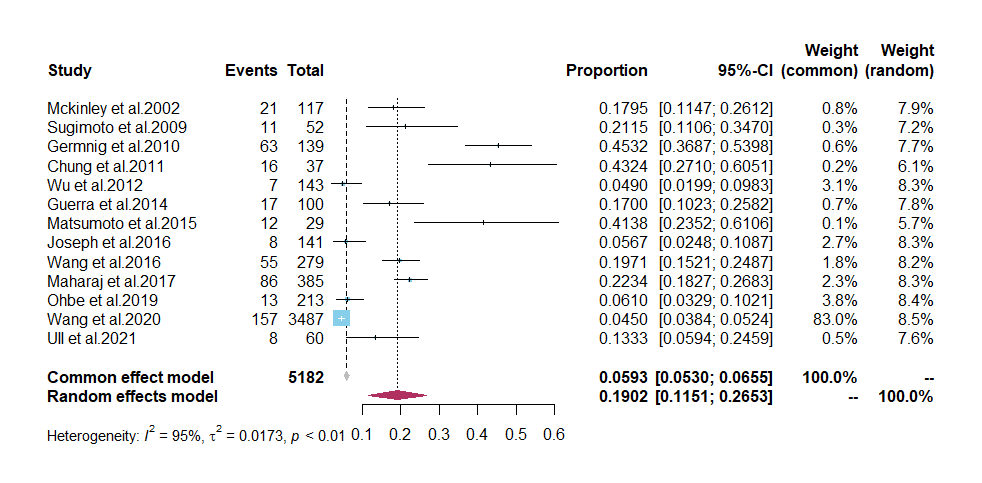
**

**Supplementary Figure S9.Publication bias**

**Egger’s test**

**
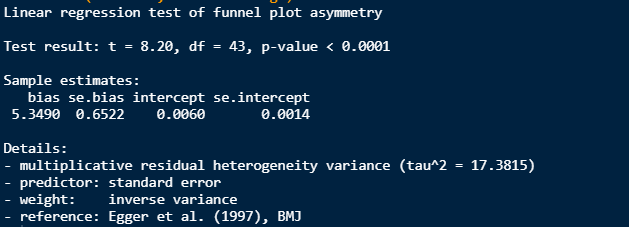
**

**Supplementary Table S1: Risk of bias assessment**

| Q2 | Q3 | Q4 | Q5 | Q6 | Q7 | Q8 | Q9 | Total (%) | Risk of Bias |
| --- | --- | --- | --- | --- | --- | --- | --- | --- | --- |
| Y | U | Y | Y | Y | Y | Y | U | 66.67 | MODERATE |
| Y | Y | Y | Y | Y | Y | Y | U | 77.78 | LOW |
| Y | Y | Y | Y | Y | Y | Y | U | 88.89 | LOW |
| Y | Y | Y | Y | Y | U | Y | U | 66.67 | MODERATE |
| Y | U | Y | Y | Y | Y | Y | U | 66.67 | MODERATE |
| Y | U | N | Y | Y | Y | Y | U | 55.56 | MODERATE |
| Y | U | N | Y | Y | U | Y | U | 44.45 | HIGH |
| Y | U | Y | Y | U | U | Y | U | 44.45 | HIGH |
| Y | U | Y | Y | Y | Y | Y | U | 66.67 | MODERATE |
| Y | Y | Y | Y | U | U | Y | U | 66.67 | MODERATE |
| Y | Y | Y | Y | Y | Y | Y | U | 88.89 | LOW |
| Y | U | Y | Y | Y | U | Y | U | 66.67 | MODERATE |
| Y | U | Y | Y | Y | U | Y | U | 55.56 | MODERATE |
| Y | U | Y | Y | Y | U | Y | U | 55.56 | MODERATE |
| Y | N | Y | Y | Y | Y | Y | U | 77.78 | LOW |
| Y | Y | N | Y | Y | U | Y | U | 66.67 | MODERATE |
| Y | N | Y | Y | Y | Y | Y | U | 77.78 | LOW |
| Y | Y | Y | Y | U | U | Y | Y | 77.78 | LOW |
| Y | Y | Y | Y | U | U | Y | Y | 66.67 | MODERATE |
| Y | Y | Y | Y | Y | Y | Y | U | 88.89 | LOW |
| Y | N | Y | Y | Y | Y | Y | Y | 77.78 | LOW |
| Y | Y | Y | Y | U | U | Y | U | 66.67 | MODERATE |
| Y | Y | Y | Y | Y | U | Y | U | 77.78 | LOW |
| Y | U | U | Y | Y | Y | Y | U | 55.56 | MODERATE |
| Y | U | Y | Y | Y | Y | Y | U | 77.78 | LOW |
| Y | U | Y | Y | Y | Y | Y | U | 66.67 | MODERATE |
| Y | Y | Y | Y | U | U | Y | U | 66.67 | MODERATE |
| Y | U | Y | Y | U | U | Y | U | 44.45 | HIGH |
| Y | U | Y | Y | Y | U | Y | U | 55.56 | MODERATE |
| Y | U | Y | Y | Y | U | Y | U | 55.56 | MODERATE |
| Y | U | Y | Y | Y | U | Y | U | 66.67 | MODERATE |
| Y | U | Y | Y | Y | U | Y | U | 55.56 | MODERATE |
| Y | U | Y | Y | U | U | Y | U | 44.45 | HIGH |
| Y | U | Y | Y | U | U | Y | U | 44.45 | HIGH |
| Y | N | Y | Y | U | U | Y | U | 44.45 | HIGH |
| Y | U | Y | Y | Y | Y | Y | U | 66.67 | MODERATE |
| Y | U | N | Y | Y | Y | Y | U | 55.56 | MODERATE |
| Y | U | Y | Y | U | U | Y | U | 55.56 | MODERATE |
| Y | U | N | Y | Y | U | Y | U | 55.56 | MODERATE |
| Y | U | Y | Y | Y | U | Y | U | 66.67 | MODERATE |
| Y | U | Y | Y | U | U | Y | U | 44.45 | HIGH |
| Y | U | Y | Y | Y | Y | Y | U | 66.67 | MODERATE |
| Y | U | Y | Y | U | U | Y | U | 44.45 | HIGH |
| Y | N | Y | Y | Y | N | Y | U | 55.56 | MODERATE |
| Y | N | Y | Y | Y | N | Y | U | 55.56 | MODERATE |

**Supplementary eMethods:**

There were 9 items in total: (1) Was the sample frame appropriate to address the target population? (2) Were study participants sampled in an appropriate way? (3) Was the sample size adequate? (4) Were the study participants and the setting described in detail? (5) Was the data analysis conducted with sufficient coverage of the identified sample? (6) Were valid methods used for identification of the condition? (7) Was the condition measured in a standard, reliable way for all of the participants? (8) Was there an appropriate statistical analysis? (9) Was the response rate adequate? If not, was the low response rate managed appropriately?
